# Glial cells modulate the synaptic transmission of NTS neurons sending projections to ventral medulla of Wistar rats

**DOI:** 10.1002/phy2.80

**Published:** 2013-09-17

**Authors:** Daniela Accorsi-Mendonça, Daniel B Zoccal, Leni G H Bonagamba, Benedito H Machado

**Affiliations:** Department of Physiology, School of Medicine of Ribeirão Preto, University of São PauloRibeirão Preto, São Paulo, Brazil

**Keywords:** Gliotransmission, NTS neurons, tripartite synapse

## Abstract

There is evidence that sympathoexcitatory and respiratory responses to chemoreflex activation involve ventrolateral medulla-projecting *nucleus tractus solitarius* (NTS) neurons (NTS-VLM neurons) and also that ATP modulates this neurotransmission. Here, we evaluated whether or not astrocytes is the source of endogenous ATP modulating the synaptic transmission in NTS-VLM neurons. Synaptic activities of putative astrocytes or NTS-VLM neurons were recorded using whole cell patch clamp. *Tractus solitarius* (TS) stimulation induced TS-evoked excitatory postsynaptic currents (TS-eEPSCs) in NTS-VLM neurons as well in NTS putative astrocytes, which were also identified by previous labeling. Fluoracetate (FAC), an inhibitor of glial metabolism, reduced TS-eEPSCs amplitude (−85.6 ± 16 vs. −39 ± 7.1 pA, *n* = 12) and sEPSCs frequency (2.8 ± 0.5 vs. 1.8 ± 0.46 Hz, *n* = 10) in recorded NTS-VLM neurons, indicating a gliomodulation of glutamatergic currents. To verify the involvement of endogenous ATP a purinergic antagonist was used, which reduced the TS-eEPSCs amplitude (−207 ± 50 vs. −149 ± 50 pA, *n* = 6), the sEPSCs frequency (1.19 ± 0.2 vs. 0.62 ± 0.11 Hz, *n* = 6), and increased the paired-pulse ratio (PPR) values (∼20%) in NTS-VLM neurons. Simultaneous perfusion of Pyridoxalphosphate-6-azophenyl-2′,5′-disulfonic acid (iso-PPADS) and FAC produced reduction in TS-eEPSCs similar to that observed with iso-PPADS or FAC alone, indicating that glial cells are the source of ATP released after TS stimulation. Extracellular ATP measurement showed that FAC reduced evoked and spontaneous ATP release. All together these data show that putative astrocytes are the source of endogenous ATP, which via activation of presynaptic P2X receptors, facilitates the evoked glutamate release and increases the synaptic transmission efficacy in the NTS-VLM neurons probably involved with the peripheral chemoreflex pathways.

## Introduction

The peripheral chemoreceptors are essential to detect changes in the oxygen in the arterial blood (PaO_2_) and its activation by hypoxia produces respiratory, autonomic, and behavioral responses (Haibara et al. 1995, 1999; Machado 2001). Anatomical and functional evidence demonstrate that the first synapse of peripheral chemoreceptor afferents in the central nervous system is located in the *nucleus tractus solitarius* ([NTS] Finley and Katz 1992; Vardhan et al. 1993; Chitravanshi et al. 1994; Chitravanshi and Sapru 1995; Paton et al. 2001) and several studies demonstrated that the sympathoexcitatory and respiratory components of chemoreflex involve NTS neurons sending projections to the ventral medulla ([NTS-VLM neurons] Ross et al. 1985; Urbanski and Sapru 1988; Seller et al. 1990; Koshiya et al. 1993; Aicher et al. 1996).

The understanding of the neurotransmitter and neuromodulators involved in the NTS neurotransmission of peripheral chemoreceptors afferents is critical in physiological and pathophysiological conditions. Previously, we showed that the combined antagonism of ionotropic glutamate and purinergic receptors within the NTS was effective in blocking pressor and sympathoexcitatory responses to peripheral chemoreceptors activation in awake rats as well as in *in situ* preparations, respectively (Braga et al. 2007). We also demonstrated that the evoked and spontaneous excitatory neurotransmission in NTS-VLM neurons are modulated by exogenous ATP (Accorsi-Mendonça et al. 2007). Recent studies demonstrated that ATP is an important extracellular messenger involved with the bidirectional signaling in the glia–neuron interaction in the tripartite synapse, modulating the neuronal responses under physiological conditions (Araque et al. 1999; Guthrie et al. 1999; Queiroz et al. 1999; Anderson et al. 2004; Koizumi et al. 2005; Gordon et al. 2009; Ben Achour and Pascual 2010). In addition to its involvement in the synaptic transmission, ATP released by astrocytes seems to be an important mediator of chemosensory transduction under challenges produced by hypoxia or pH alterations in central neuronal systems (Erlichman et al. 2010; Gourine et al. 2010; Huckstepp et al. 2010).

Considering that (1) NTS plays a key role in the processing of afferent information from the peripheral chemoreceptors; (2) NTS presents a high density of glial cells (Pecchi et al. 2007), and that (3) astrocytes cover the NTS neurons (Tashiro and Kawai 2007), in this study we carefully evaluated the role of NTS glial cells in the ATP release and its role in the modulation of evoked and spontaneous neurotransmission in neurons integral to the peripheral chemoreflex pathways.

## Material and Methods

The experimental protocols used in this study were approved by the Institutional Ethical Committee on Animal Experimentation of the School of Medicine of Ribeirão Preto, University of São Paulo (Protocol of the Animal Ethic Committee # 070/2007).

### Microinjection of tracer into the VLM

To identify the cell bodies of NTS-VLM neurons, fluorescent tracer was microinjected into the VLM as previously described by Accorsi-Mendonça et al. (2009). Briefly, Wistar male rats (270–290 g) were anesthetized with tribromoethanol (intraperitoneal injection of 250 mg/kg; Sigma-Aldrich, Milwaukee, WI). Previous to microinjection of tracer, the VLM area was functionally identified by microinjection of l-glutamate (Willette et al. 1987). For records of arterial pressure, a femoral artery was catheterized using a catheter tube (PE50; Clay Adams, Parsippany, NJ) connected to an amplifier (Bridge Amp ML221; ADInstruments, Bella Vista, NSW, Australia), via a pressure transducer (MLT0380; ADInstruments). The signals of pulsatile arterial pressure (PAP), mean arterial pressure (MAP), and heart rate (HR) were acquired (PowerLab4/25 ML845; ADInstruments) and recorded (Chart Pro software; ADInstruments). Subsequently, the animals were placed in a stereotaxic apparatus (David Kopf, Tujunga, CA) and a little hole was drilled in the skull for nanoinjections into the VLM using 33G needle, in accordance with the coordinates of the atlas by Paxinos and Watson (1986): 1.8 mm lateral to the midline, 3.2 mm caudal to the lambda, and 10.2 mm below the skull surface). The needle used for nanoinjections (33 gauge, Small Parts, Miami Lakes, FL) was connected by a polyethylene tube (PE-10; Clay Adams) to a 1-μL syringe (Hamilton, Reno, NV). Initially, l-glutamate (20 mmol/L/100 nL) was microinjected and a pressor response was used as a functional index to characterize the right VLM site. In the sequence, the bidirectional membrane tracer 1,1′-dioctadecyl-3,3,3′,3′- tetramethylindocarbocyanine perchlorate ([DiI, 0.5% in ethanol] Invitrogen, Carlsbad, CA) or the retrograde tracer Green Retrobeads™IX (LumaFluor Inc., New York, NY) were microinjected into the same site. After injections into the VLM the needle was removed, skin was sutured, the catheter was removed from the femoral artery, which wall was sutured and the animal treated with an antibiotic intramuscularly (Pentabiotic Veterinarian; Fort Dodge Saúde Animal Ltda., Campinas, SP, Brazil). The animals were allowed to recovery from the surgical procedures for at least 1 week. Before the electrophysiological experiments the site of microinjections was examined in freshly prepared brainstem slices (for details see below) using epifluorescence illumination. Slices in which the site of injections was not properly located were not used.

### Brainstem slices preparation

At the day of experiment the animals were deeply anesthetized with sodium pentobarbital ([ip, 0.05 g/kg] Cristália Produtos Químicos e Farmacêuticos Ltda., Itapira, SP, Brazil) and decapitated. The brain was rapidly removed and submerged in modified ice-cold (4°C) artificial cerebrospinal fluid (sucrose-aCSF) containing the following (in mmol/L): 75 sucrose, 87 NaCl, 2.5 KCl, 7 MgCl_2_, 1.25 NaH_2_PO_4_, 25 NaHCO_3_, 25 glucose, and 0.2 CaCl_2_, with osmolality of ∼ 330 mOsm/Kg H_2_O and pH 7.4 when bubbled with 95% O_2_ and 5% CO_2_. Brainstem transversal slices (250 μm thick) were cut using an oscillating slicer (Vibratome 1000 plus; Vibratome, St. Louis, MO) and kept in sucrose-aCSF at 35°C for 30 min. Thereafter, the slices were kept at room temperature (RT, 23–25°C) in normal-aCSF containing (mmol/L): 125 NaCl, 2.5 KCl, 1 MgCl_2_, 1.25 NaH_2_PO_4_, 25 NaHCO_3_, 25 glucose, and 2 CaCl_2_, with osmolality of ∼300 mOsm/Kg H_2_O and pH 7.4 when bubbled with 95% O_2_ and 5% CO_2_. Before recordings, a single slice was placed into the recording chamber, held in place with a nylon thread and continuously perfused with aCSF at a flow of approximately 2–3 mL/min at RT.

Cells (neurons or putative astrocytes) in brainstem slice were visualized using infrared and differential interference contrast (IR-DIC) microscopy (Olympus BX51WI; Olympus, Tokyo, Japan) through a 40× water immersion objective (LUMPlain F1-IR; Olympus) and a charge-coupled device camera (C7500-50; Hamamatsu, Iwata-City, Japan). After visualizing the neuron in the IR-DIC optic, the presence of fluorescence tracer was confirmed using the epifluorescence illumination and the fluorescence and IR-DIC images were superimposed to identify labeled NTS-VLM neurons.

### Whole cell patch-clamp recordings

Whole cell recordings were made with patch pipettes pulled from thick-walled borosilicateglass capillaries (Sutter Instruments, Novato, CA), using a puller (P-97; Sutter Instruments). The patch pipettes were filled with an internal solution containing (mmol/L): 130 KCl, 5 NaCl, 1 MgCl2, 3 Mg-ATP, 0.2 Na-ATP, 5 ethylene, glycol-bis(β-aminoethylether)-N,N,N′,N′-tetraacetic acid (EGTA), 10 N-2-hydroxy-ethylpiperazine-N′-2-ethanesulfonic acid (HEPES), and 0.005 lidocaine N-ethyl bromide (QX314), osmolality of ∼310 mOsm/Kg H_2_O and pH 7.4 adjusted with KOH (Chen and Bonham 2005). The final resistance of pipette ranged from 4 to 8 MΩ. The whole cell configuration was obtained, signals were acquired using an amplifier (Axopatch 200B; Axon Instruments, Sunnyvale, CA) connected to a data acquisition system (Digidata 1440A; Axon Instruments) and recorded in a microcomputer using software (Clampex, pClamp version 10; Axon Instruments). Based in the Nyquist theorem (Sampling theorem) the data were filtered (low-pass filter – 2 kHz) and acquired at least twice the filter value (10 kHz), as previously described (Shigetomi and Kato 2004; Chen and Bonham 2005; Kline et al. 2007; Zhang et al. 2008, 2009). Series resistance (<30 MΩ) was checked regularly during the experiments and cells with large variations in series resistances were discarded. The cells were held at –70 mV and recordings started 5 min after establishing the whole cell configuration. The input resistance was determined from responses to short hyperpolarizing voltage pulse (−5 mV, 20 msec).

The putative NTS astrocytes were identified based upon the electrophysiological phenotype: no action potential spiking behavior and absence of voltage-gated Na^+^ currents (Volterra and Meldolesi 2005; Panatier et al. 2011). Absence of action potential was verified in current-clamp configuration with injection of depolarized currents with 12.5 pA increments during 2 sec. The absence of voltage-dependent Na^+^ channels in NTS astrocytes was verified in voltage-clamp configuration with membrane holding at −70 mV. The currents were induced by voltage steps in pulses from −100 to −40 mV, with 10 mV increments and duration of 700 msec (Zhou et al. 2006). In order to have a better characterization of putative NTS astrocytes we also used in some experiments the dye Sulforhodamine-101 (SR-101), which is taken up by protoplasmic astrocytes (Nimmerjahn et al. 2004). Briefly, NTS slices were incubated in sucrose-ACSF solution containing SR-101(0.7 μmol/L) at 34–35°C for 25 min, followed by 15 min in dye free sucrose-ACSF and then maintained at room temperature in ACSF (McDougal et al. 2011; Huda et al. 2013).

Evoked excitatory postsynaptic currents (eEPSCs) in neurons or glial cells were generated by electrical stimulation of ipsilateral *Tractus solitarius* (TS) using a concentric bipolar microelectrode (Frederick Haer & Co., Bowdoin, ME) connected to an isolated stimulator (DS2A; Digitimer, Letchworth Garden City, U.K.). The intensity used for TS stimulation (0.1 msec duration, 0.07 Hz) was that required to produce maximal eEPSC amplitude. The peak amplitude of TS-eEPSC was measured using software (Clampfit, pClampversion 10; Axon Instruments).

In neuronal recordings we also applied the paired-pulse ratio (PPR) protocol and the amplitudes of two consecutive TS-eEPSCs (P1 and P2, respectively) with interstimulus interval of 100 msec were analyzed. Usually PPR protocol evokes two TS-eEPSCs in NTS neurons and the first TS-eEPSC presents larger amplitude when compared with the second. NTS-VLM neurons with the first peak smaller than the second was not used in our analysis. The analysis of PPR value (P2/P1) in the presence of a certain drug reveals possible pre- and/or postsynaptic effects of this treatment (Kline et al. 2002; Sekizawa et al. 2003).

The data are expressed as mean ± standard error (SEM). Statistical significance (*P* < 0.05) was determined by paired or unpaired Student's *t*-test or analyses of variance (ANOVA) one-way with Bonferroni posttest using software (GraphPad Prism version 4; GraphPad Prism, USA). Kolmogorov–Smirnov (KS) test (http://www.physics.csbsju.edu/stats/KS-test.n.plot_form.html) was used to analyze the distribution of amplitude.

### Extracellular ATP measurements

Extracellular ATP was evaluated by bioluminescence method using luciferase-luciferin test as previously described (Cotrina et al. 1998; Zhang et al. 2003). NTS coronal slices (250 μm thickness) were placed in a perfusion chamber (1 mL) containing aCSF bubbled with 95% O_2_ and 5% CO_2_. The TS was stimulated by 10 trains of 5 stimuli (50 V, 0.1 msec duration, 10 Hz during the train and 0.33 Hz between trains) using a concentric bipolar microelectrode (Frederick Haer & Co.) connected to an isolated stimulator (DS2A; Digitimer). One minute after TS stimulation, sample of liquid perfusion (50 μL) was collected from the NTS region near the surface of slice and adjacent to the central channel. The perfusion was stopped during the TS stimulation and sampling, but the bubbling of the carbogenic mixture (95% O_2_ and 5% CO_2_) in the chamber was maintained. The sample of 50 μL was added into 50 μL of ATP Assay Mix (ATP bioluminescent assay kit; Sigma, Milwaukee, WI) and the luminescence was measured in triplicate by luminometer (Sirius Single tube Luminometer; Berthold Technology, Bad Wildbad, Germany). The luminescence observed in aCSF sample obtained in presence of a slice without any stimulation of TS was considered as background. In some experiments the slices were perfused during 20 min with aCSF solution containing Cd^2+^ (50 μmol/L, to block the voltage-dependent Ca2+ channels), fluoracetate (FAC; 1 mmol/L), 6,7-dinitroquinoxaline-2,3-dione (DNQx; 10 μmol/L, to antagonize non-NMDA receptors). These data are expressed as individual values and the statistical significance (p<0.05) of mean ± SEM was determined by paired Student t-test using software (GraphPad Prism version 4 – GraphPad Prism).

### Drugs

The slice in the recording chamber was perfused with aCSF by a gravity-driven perfusion system (flow: 2–3 mL/min). All drugs were diluted in the aCSF solution and the flow throughout the recording chamber containing the slices was regulated by a 6-valve solenoid system (VC-6; Warner Instruments, Hamden, CT). Pyridoxalphosphate-6-azophenyl-2′,5′-disulfonic acid (iso-PPADS) and were obtained from Tocris (Ellisville, MO). DNQx, QX314, (FAC), sulforhodamine 101, and bicuculline were obtained from Sigma. Bicuculline, a γ-aminobutyric acid A receptor subtype antagonist (GABA_A_), was dissolved in dimethylsulfoxide ([DMSO] Sigma), and the final concentration of DMSO in the aCSF bath was in the range of 0.1%.

## Results

### Putative NTS astrocytes recordings

**Figure 1:**
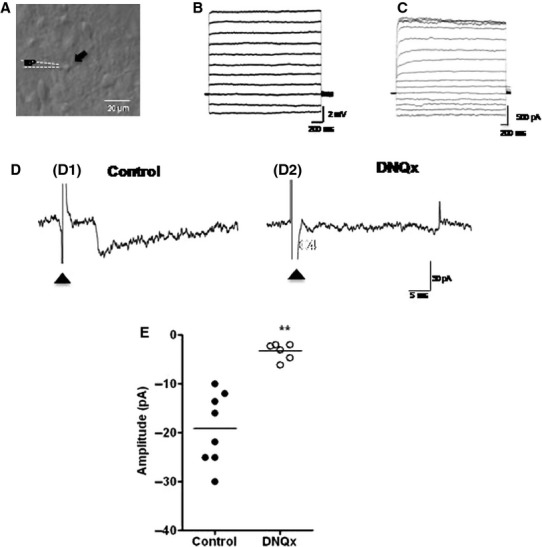
TS stimulation induces current in *nucleus tractus solitarius (*NTS) astrocytes. (A) Photomicrography showing one putative astrocyte (arrow) on the surface of the NTS slice viewed on infrared and differential interference contrast (IR-DIC) optic, dashed line: recording pipette (RP); (B) Representative tracings showing the voltage x current relationship of one NTS astrocyte, note the absence of action potential; (C) Representative tracings showing the current × voltage relationship of one NTS astrocyte, note the absence of sodium voltage-dependent currents; (D) Representative tracings showing the evoked current in one NTS astrocyte after TS stimulation before (D1) and after DNQx perfusion (D2), arrow: the stimulus artifact; (E) Mean amplitude of evoked current in NTS astrocytes (*n* = 5) before and after DNQx perfusion, *P < 0.05.

Initially we verified the sensitivity of putative NTS astrocytes to TS stimulation. For this purpose we recorded cells smaller than neurons under IR-DIC microscopy (Fig. 1A) and the putative astrocytic electrophysiological profile was confirmed by the absence of spikes after injection of positive current (Fig. 1B) and voltage-gated Na^+^ currents (Fig. 1C). The SR101 dye was also used in some experiments as a second experimental approach for better identification of putative astrocytes (Nimmerjahn et al. 2004). After TS stimulation, the putative NTS astrocytes presented small-evoked inward current (−19.18 ± 2.6 pA, *n* = 8, Fig. 1D and E), showing that putative astrocytes in the NTS are sensitive to TS stimulation. In relation to the success/failure rate the putative astrocytes presented a higher failure rate after TS stimulation when compared to the neurons (16 ± 6% vs. 4.2 ± 2%, astrocytes [*n* = 6] and neurons [*n* = 12], respectively; unpaired *t-*test *P* = 0.036).

Considering the evidence by McDougal et al. (2011) that the increase in intracellular calcium concentration in NTS astrocytes after stimulation of vagal afferents was mediated by α-amino-3-hydroxy-5-methyl-4-isoxazolepropionic acid receptors (AMPA receptors), we investigated if the evoked current observed in putative astrocytes was also mediated by AMPA receptors stimulation. The presence of DNQx, a selective non-NMDA receptor antagonist, reduced the evoked activity in putative NTS astrocytes (−19.18 ± 2.6 vs. −3.31 ± 0.7 pA, *P* = 0.006; Fig. 1D and E), showing a key role for AMPA receptors in the putative astrocytes responses to TS stimulation.

### Astrocytic modulation on evoked and spontaneous activities of NTS-VLM neurons

Considering that TS stimulation induces glutamate release and produces an TS-EPSCs in the NTS-VLM neurons (Accorsi-Mendonça et al., 2007), and also that astrocytes are around the synaptic cleft of NTS neurons (Tashiro and Kawai 2007) we induced the putative astrocytes inhibition to evaluate their role in the response of NTS-VLM neurons after TS stimulation.

**Figure 2:**
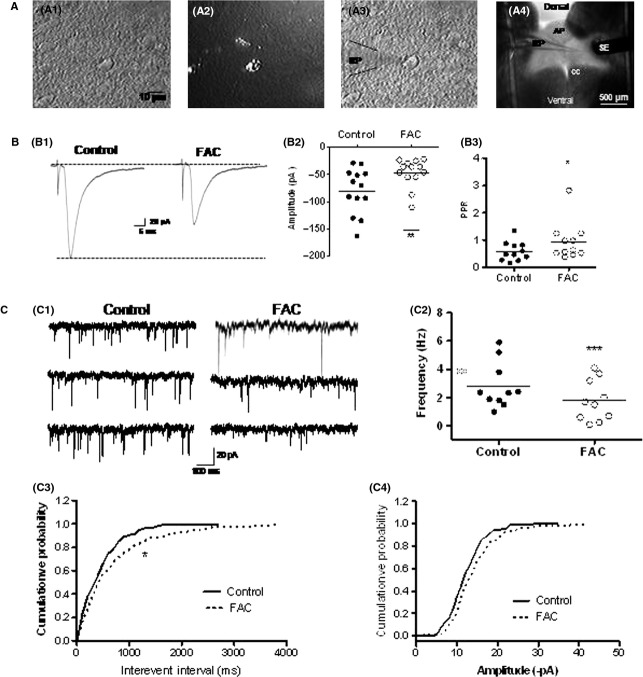
Astrocytic modulation on *nucleus tractus solitarius-*ventral medulla (NTS-VLM) neurons. (A1) Photomicrography showing one neuron on the surface of the slice, in the NTS, viewed on infrared and differential interference contrast (IR-DIC) optic; (A2) Photomicrography of the same NTS neuron presented in panel A1, which is a labeled cell, viewed under fluorescence; (A3) Photomicrography of same NTS neuron presented in Panels A1 and A2 viewed on IR-DIC optic in presence of the recording pipette (RP-dashed line); (A4) Photomicrography of a coronal brainstem slice containing the NTS and showing the position of the RP in relation to the stimulating electrode (SE) placed on the tractus solitarius (TS); (B1) Representative tracings of TS-evoked excitatory postsynaptic currents (TS-eEPSCs) from one representative NTS-VLM neuron before and during fluoracetate (FAC) perfusion (1 mmol/L-20 min); (B2) Mean of TS-eEPSCs amplitude before and after FAC perfusion (*n* = 12); (B3) Mean of paired pulsed ratio (PPR) before and after FAC perfusion (*n* = 11); (C1) Representative tracings of sEPSCs from one representative NTS-VLM neuron before and during FAC perfusion (1 mmol/L-20 min); (C2): Mean of sEPSCs frequency before and after FAC perfusion (*n* = 10); (C3) Cumulative probability of interevent interval of sEPSCs from one representative NTS-VLM neuron before and after FAC perfusion; (C4) Cumulative probability of sEPSCs amplitude from one representative NTS-VLM neuron before and after FAC perfusion; *P < 0.05; **P < 0.001; ***P < 0.0001.

Fluoracetate, an astrocytic metabolic inhibitor (Fonnum et al. 1997), was used in the bath perfusion to block the astrocytic activity. FAC (1 mmol/L, 20 min) reduced the TS-eEPSCs amplitude (−80.23 ± 16 vs. −46.4 ± 7 pA, *n* = 13, *P* = 0.0045; Figs. 2A and B), but did not change the rise time (2.2 ± 0.3 vs. 2.4 ± 0.26 msec, *n* = 13, *P* = 0.44), decay time (9.1 ± 1.3 vs. 8.6 ±1.3 msec, *n* = 13, *P* = 0.53), or half-width (5.6 ± 0.64 vs. 5.5 ± 0.7 msec, *n* = 13, *P* = 0.73) of evoked current of NTS-VLM neurons. FAC also increased the PPR (0.56 ± 0.1 vs. 0.94 ± 0.21, *n* = 11, *P* = 0.03; Fig. 1B), indicating that astrocytic inhibition affects the evoked neurotransmission of NTS-VLM neurons by a presynaptic mechanism (Kline et al. 2002; Sekizawa et al. 2003).

To test the possibility that the reduction of TS-eEPSCs amplitude induced by FAC was due to a depletion of the glutamine source (Berg-Johnsen et al. 1993; Belanger et al. 2011), we also evaluated the effects of FAC on the evoked current in the presence of glutamine (200 μmol/L). The reduction of TS-eEPSCs amplitude produced by FAC was similar in the presence or absence of glutamine (FAC: 0.59 ± 0.09% *n* = 12; FAC + glutamine: 0.56 ± 0.09%, *n* = 12, *P* = 0.86), indicating that the reduction of TS-eEPSCs amplitude by FAC in NTS-VLM neurons was not dependent on the glutamine availability.

The role of putative NTS astrocytes in the spontaneous neurotransmission in NTS-VLM neurons was also evaluated. The astrocytic inhibition using FAC reduced the frequency of spontaneous events ([sEPSCs] 2.8 ± 0.5 vs. 1.8 ± 0.46 Hz, *n* = 10, *P* = 0.0003, Fig. 2C) but did not change the amplitude (14.44 ± 2.3 vs. 13.7 ± 2 pA, *n* = 10, *P* = 0.59) or half-width of spontaneous events (3.1 ± 0.4 vs. 2.8 ± 0.4 msec, *n* = 10, *P* = 0.3), suggesting that putative NTS astrocytes inhibition affects the sEPSCs by a presynaptic mechanism. Therefore, the inhibition of putative astrocytes decreases the evoked and spontaneous neurotransmission in NTS-VLM neurons by a presynaptic mechanism.

### Purinergic modulation of evoked and spontaneous activities of NTS-VLM neurons

**Figure 3:**
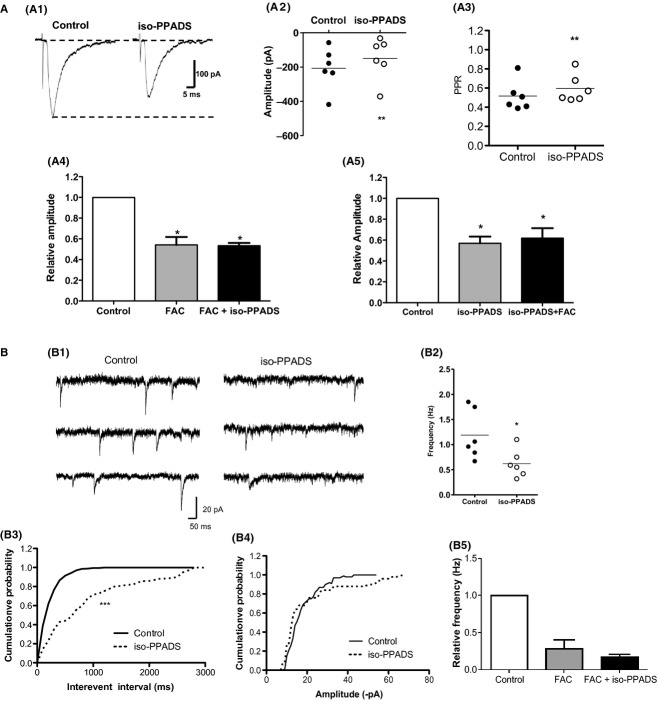
Purinergic modulation on *nucleus tractus solitarius-*ventral medulla (NTS-VLM) neurons.(A1) Representative tracings of TS-eEPSCs from one representative NTS-VLM neuron before and after Pyridoxalphosphate-6-azophenyl-2′,5′-disulfonic acid (iso-PPADS) perfusion (100 μmol/L-20 min); (A2) Mean of TS-eEPSCs amplitude before and after iso-PPADS perfusion (*n* = 6); (A3) Mean of paired pulsed ratio (PPR) before and after iso-PPADS perfusion (*n* = 6); (A3) Mean of TS-eEPSCs amplitude before and after FAC and FAC + iso-PPADS perfusion (*n* = 6); (A4) Mean of TS-eEPSCs amplitude before and after iso-PPADS and iso-PPADS + FAC perfusion (*n* = 7); (B1) Representative tracings of sEPSCs from one representative NTS-VLM neuron before and during iso-PPADS perfusion (100 μmol/L, 20 min); (B2) Mean of sEPSCs frequency before and after iso-PPADS perfusion (*n* = 6); (B3) Cumulative probability of interevent interval of sEPSCs from one representative NTS-VLM neuron before and after iso-PPADS perfusion; (B4) Cumulative probability of sEPSCs amplitude from one representative NTS-VLM neuron before and after iso-PPADS perfusion; (B5) Relative frequency of sEPSCs before and after FAC and FAC + iso-PPADS perfusion; *P < 0.05; **P < 0.001; ***P < 0.0001.

Considering that astrocytes are an important source of endogenous ATP (Guthrie et al. 1999; Queiroz et al. 1999; Anderson et al. 2004; Koizumi et al. 2005; Hamilton and Attwell 2010) and also that ATP affects the neurotransmission in NTS-VLM neurons (Accorsi-Mendonça et al. 2009), here we evaluated the role of putative astrocytes as the source of ATP that modulates the sEPSCs and TS-eEPSCs recorded in NTS-VLM neurons. To verify the role of endogenous ATP, we applied iso-PPADS, a P2 receptor antagonist in the bath perfusion (Khakh et al. 2001). Iso-PPADS (100 μmol/L – 20 min) decreased the amplitude of TS-eEPSCs (−223 ± 53 vs. −156 ± 51 pA, *n* = 6, *P* = 0.0033; Fig. 3A), increased the PPR value (0.52 ± 0.06 vs. 0.59 ± 0.06, *n* = 6, *P* = 0.048; Fig. 3A), but did not change the rise time (3 ± 0.34 vs. 2.8 ± 0.15 msec, *n* = 6, *P* = 0.56), decay time (15.2 ± 1.16 vs. 16 ± 1.14 msec, *n* = 6, *P* = 0.40), or half-width (9.6 ± 1 vs. 10.28 ± 1 msec, *n* = 6, *P* = 0.11) of evoked currents, indicating that TS stimulation releases ATP, which via P2X receptors, affects the TS-eEPSCs in NTS-VLM neurons by presynaptic mechanisms.

On the next step we evaluated whether putative astrocytes release ATP in response to TS stimulation. The astrocytic metabolism was inhibited by FAC, which reduced the TS-eEPSCs amplitude and in the sequence the slices were overperfused with iso-PPADS to antagonize P2 receptors. After the astrocytic inhibition by FAC, the iso-PPADS produced no additional change in the TS-eEPSCs (FAC: 54 ± 8% vs. FAC + iso-PPADS: 53 ± 3%, *n* = 6; *P* = 0.26; Fig. 3A), suggesting that putative NTS astrocytes release ATP in response to TS stimulation.

In another series of experiments the slices were perfused with iso-PPADS to antagonize P2 receptors and in the sequence the astrocytic activity was inhibited by adding FAC in the bath during iso-PPADS perfusion. The overperfusion with FAC produced no additional changes on the effects of iso-PPADS (iso-PPADS: 57 ± 6% vs. iso-PPADS + FAC: 62 ± 9%, *n* = 7, *P* = 0.11; Fig. 3A), demonstrating that after purinergic receptors antagonism, the putative NTS astrocytes are not affecting the modulation of TS-eEPSCs. Taken together, these data are consistent with the concept that TS stimulation induces ATP release by putative astrocytes, which activate P2X receptors located at the presynaptic terminals facilitating the glutamate release onto the synapses of NTS-VLM neurons.

Spontaneous neurotransmission in NTS-VLM neurons was under influence of purinergic modulation, as iso-PPADS reduced the frequency (1.19 ± 0.2 vs. 0.62 ± 0.11 Hz, *n* = 6, *P* = 0.0403; Fig. 3B), but produced no effect on the amplitude (19.4 ± 2.5 vs. 16 ± 2.1 pA, *n* = 6, *P* = 0.27) or half-width of spontaneous events (3.07 ± 0.24 vs. 2.7 ± 0.32 msec, *n* = 6, *P* = 0.31). We also evaluated the effect of astrocytic inhibition and purinergic receptors antagonism on spontaneous currents. The presence of FAC reduced the frequency (5.3 ± 1.5 vs. 1.14 ± 0.33 Hz, *n* = 5; Fig. 3B5), but produced no effect on the amplitude (16.2 ± 1.8 vs. 15 ± 1.4 pA) or half-width of spontaneous events (2.3 ± 0.2 vs. 2.21 ± 0.5 msec). Subsequently, the overperfusion with iso-PPADS produced no additional effect on the sEPSCs (Frequency: 0.93 ± 0.23 Hz, Amplitude: 13.5 ± 1.4 pA, Half-width: 2.24 ± 0.35 msec; *n* = 5), indicating that after astrocytic inhibition the purinergic receptors are not activated (Fig. 3B5). These results show that ATP is spontaneously released by putative astrocytes in the NTS.

### Extracellular ATP evaluation

**Figure 4:**
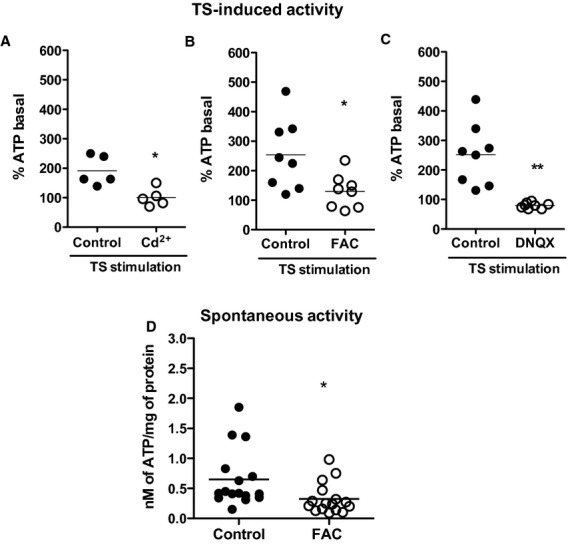
Extracellular ATP evaluation. (A) Changes in extracellular ATP in response to TS stimulation in control situation or in the presence of Cd^2+^ (50 μmol/L) in the bath solution (*n* = 5); (B) Changes in extracellular ATP in response to TS stimulation in control situation or in the presence of fluoracetate (FAC; 1 mmol/L) in the bath solution (*n* = 8); (C) Changes in extracellular ATP in response to TS stimulation in control situation or in the presence of DNQx (20 μmol/L) in the bath solution (*n* = 8); (D) Changes in extracellular ATP basal level in control situation or in the presence of FAC in the bath solution (*n* = 16); *P < 0.05; **P < 0.05.

To evaluate that in response to TS stimulation putative NTS astrocytes really release ATP, we used the bioluminescence method to measure the extracellular ATP in the bath perfusion. The data showed that trains of TS stimulation significantly increases the extracellular ATP in relation to the baseline (143 ± 27%, *n* = 29, Fig. 4). Cd^2+^ (50 μmol/L) was used to block the voltage-dependent Ca^2+^ channels and prevents the synaptic transmission. The presence of Cd^2+^ inhibited the increase of ATP in response to TS stimulation (0.8 ± 0.01%, *n* = 5), indicating that the increase in the extracellular ATP was dependent on synapses (Fig. 4A).

To verify that putative NTS astrocytes are the source of ATP released by TS stimulation, we evaluated the increase in extracellular ATP after TS stimulation during astrocytic inhibition. In the presence of FAC the extracellular ATP, measured by the bioluminescence method, was significantly reduced (30 ± 0.2%, *n* = 8, Fig. 4B), demonstrating that putative NTS astrocytes releases ATP in response to TS stimulation. Recently, McDougal et al. (2011) demonstrated that vagal afferent stimulation activates NTS astrocytes, via AMPA receptors, and increases the intracellular calcium concentration. Based upon their finding, we also evaluated the role of AMPA receptors in astrocytic transmission induced by TS stimulation. Bath application of DNQX (10 μmol/L), a selective non-NMDA receptor antagonist, prevented the increase of extracellular ATP in response to TS stimulation (0.09 ± 0.004%, *n* = 6; Fig. 4C), supporting the concept that after TS stimulation, glutamate activates AMPA receptors, which induces ATP releases.

The involvement of putative NTS astrocytes on spontaneous ATP releases was also confirmed using measurement of extracellular ATP basal level, as the presence of FAC (1 mmol/L) decreased the spontaneous ATP release in NTS slices (0.65 ± 0.12 vs. 0.33 ± 0.06 mmol/L ATP/mg of protein, *n* = 16; Fig. 4D).

## Discussion

The overall data of this study support the concept that endogenous ATP, released by putative NTS astrocytes, plays a facilitatory role on evoked and spontaneous glutamate release, via P2X receptors activation, onto NTS-VLM neurons, which probably are integral to the chemoreflex pathways. The activation of peripheral chemoreflex produces sympathoexcitatory/pressor responses, respiratory and behavioral reflex adjustments (Machado et al. 1997). Regarding the pressor response, different studies have shown that this sympathoexcitatory component involves neuronal pathways from NTS to VLM (Seller et al. 1990; Koshiya et al. 1993). However, there is no previous experimental evidence that astrocytes modulate the synaptic transmission of NTS-VLM neurons.

As NTS presents a density of glial cells higher than other brainstem areas (Pecchi et al. 2007) and there is ultrastructural evidence of tripartite synapses in the NTS (Tashiro and Kawai 2007; Chounlamountry and Kessler 2011), in this study, we documented that putative astrocytes in the NTS modulate the spontaneous neuronal transmission and the synaptic activity in response to TS stimulation. For this purpose, we patched putative NTS astrocytes and evaluated their sensitivity to TS stimulation. The TS stimulation produced an inward current in NTS glial cells, which was blocked by DNQx, a glutamatergic inotropic receptor antagonist. These data are in accordance with McDougal et al. (2011), which showed that NTS astrocytes are activated by TS stimulation orexogenous AMPA application, as indicated by increases in astrocytic intracellular calcium concentrations. Therefore, our findings show that putative NTS astrocytes are sensitive to TS stimulation and the observed EPSCs are produced via activation of non-NMDA receptor.

We also evaluated the modulatory role of putative astrocytes on evoked and spontaneous transmission in NTS-VLM neurons. To inhibit the astrocytic activity we used FAC, an inhibitor of glial metabolism (Fonnum et al. 1997). FAC in the bath produced a reduction in TS-eEPSCs amplitude and sEPSCs frequency. These data documented the role of putative astrocytes on the modulation of synaptic transmission of NTS-VLM neurons. FAC in the bath also increased the PPR value and produced no changes on sEPSCs amplitude or half-width, confirming that this inhibitor does not have effect on NTS-VLM neurons and acts at presynaptic level, that is, via astrocytes, the inhibition of which precludes the gliotransmitter to modulate the neurotransmission.

Several studies demonstrated that astrocytes release ATP (Caciagli et al. 1988; Guthrie et al. 1999; Coco et al. 2003; Newman 2003a,b; Zhang et al. 2003; Ben Achour and Pascual 2010; Hamilton and Attwell 2010; Torres et al. 2012) and also that NTS neurons are responsive to exogenous ATP (Ergene et al. 1994; Barraco et al. 1996; Scislo et al. 1997; de Paula et al. 2004; Antunes et al. 2005a,b; Accorsi-Mendonça et al. 2009). In previous study we documented that glutamate and ATP present a complex interaction in the NTS during peripheral chemoreceptor activation and they seem to act as cotransmitters of the sympathoexcitatory component of chemoreflex at the NTS level (Braga et al. 2007). In this study, we tested the hypothesis that putative NTS astrocytes release ATP as a gliotransmitter affecting the neurotransmission in NTS-VLM neurons involved with the chemoreflex pathways and the data demonstrated that endogenous ATP facilitates the evoked and spontaneous glutamate release onto NTS-VLM neurons, since iso-PPADS, a P2X receptor antagonist, decreased the amplitude of TS-eEPSC and the frequency of sEPSCs. These findings clearly indicate that endogenous ATP contributes to the excitatory neurotransmission, via activation of P2X receptor subtype.

The PPR value before and after addition of P2X receptor antagonist in the bath perfusion was evaluated to verify if the effect of iso-PPADS on NTS-VLM neurons was due to pre- or postsynaptic mechanisms. The iso-PPADS increased the PPR of TS-eEPSCs and produced no changes in the amplitude or half-width of sEPSCs; these parameters indicate that iso-PPADS affects the evoked and spontaneous neurotransmission by presynaptic mechanisms (Voronin 1993; Kline et al. 2002; Sekizawa et al. 2003). It is important to note that this functional evidence of presynaptic location for P2 receptors is in agreement with previous studies by Llewellyn-Smith and Burnstock (1998) showing that most of P2X_3_ receptor immunoreactive in the NTS is located in axons of TS.

Considering that FAC inhibits most of astrocytic activities and not only the release of gliotransmitters, we could not rule out the possibilities that FAC was also affecting other astrocytic activities such as, uptake of neurotransmitters, maintenance of pH, and extracellular potassium, as well, the supply of glutamine to presynaptic terminals (Szerb and Issekutz 1987; Swanson and Graham 1994; Fonnum et al. 1997; Largo et al. 1997; Magistretti and Chatton 2005). To be sure that the depression of TS-eEPSCs induced by FAC was not due to a depletion of the glutamine, a specific experimental protocol was performed in the presence of glutamine and the data indicated that the reduction of evoked synaptic transmission by FAC was not secondary to the lack of glutamine. This observation is in accordance with previous study demonstrating that glutamate release probability in CA3–CA1 hippocampal synapses is modulated by astrocytes independently of glutamine availability (Bonansco et al. 2011).

Using different experimental protocols, we demonstrated that endogenous ATP from putative NTS astrocytes modulates the evoked and spontaneous excitatory transmission in NTS-VLM neurons. Simultaneous perfusion of iso-PPADS and FAC produced reduction in TS-eEPSCs amplitude or sEPSCs frequency, similar by that observed in the presence of iso-PPADS or FAC alone, indicating that putative astrocytes are the unique source of ATP in response to TS stimulation or under resting conditions.

Based upon these findings we suggest that during TS stimulation, putative NTS astrocytes cells are activated via non-NMDA receptors. After stimulation putative astrocytes release ATP, which acts on P2X_2_ receptors located at the presynaptic terminals, facilitating the glutamate release onto NTS-VLM neurons. In order to confirm the electrophysiological data that putative astrocytes release ATP in response to TS stimulation, we also evaluated the extracellular ATP by bioluminescence method. In experimental conditions similar to that used for electrophysiological experiments, the TS stimulation produced an increase in extracellular ATP, which was blocked in the presence of Cd^2+^. Therefore, the increase in ATP was dependent of exocytose and not related to current spread from the stimulating electrode or direct mechanical stimulation of putative astrocytes. When FAC was added to the bath perfusion, it was observed a significant reduction in the ATP release induced by TS stimulation, documenting that putative astrocytes are the source of ATP.

Considering that AMPA receptors are present in astrocytes in the NTS (McDougal et al. 2011), we also evaluated the effect of non-NMDA receptor antagonist on ATP release. DNQx blocked the increase in extracellular ATP in response to TS stimulation, confirming the involvement of non-NMDA receptors in the release of ATP by putative astrocytes. In addition, we demonstrated that FAC decreased the spontaneous release of ATP. Therefore, using a bioluminescence method, we confirmed that putative NTS astrocytes trigger the ATP release in both spontaneous and evoked neurotransmission. In pathophysiological conditions such as hypoxia in victims of sudden infant death syndrome, there is an increase in the astrocytic density at the NTS level, without changes in the neuronal density (Biondo et al. 2004). Moreover, Hermann et al. (2009) suggested that NTS astrocytes have a direct effect on the autonomic control of the gut during bleeding or trauma. In these cases NTS astrocytes expressing the PAR1 receptors, activated by thrombin, can release glutamate and increase the neuronal excitability of NTS neurons, affecting the gastric vago-vagal reflex and the control of the gastric motility (Hermann et al. 2009).

We conclude that the response to TS-stimulation involves an interaction between putative astrocytes and neurons to facilitate the excitatory synaptic transmission in NTS-VLM neurons. This interaction involves stimulation of putative astrocytes, via AMPA receptors, to induce ATP release and activation P2Xreceptors located at presynaptic terminal onto NTS-VLM neurons. This complex interaction between putative astrocytes and NTS-VLM neurons may play a key role in the processing of afferent information from peripheral chemoreceptors in the brainstem. Although the acute inhibition of putative astrocytes in the NTS in *in situ* experiments (working heart–brainstem preparation) did not affect the baseline sympathetic and respiratory activities (Costa et al. 2013), indicating that astrocytic activity is not affecting the respiratory and sympathetic outflow, we cannot exclude the possibility that astrocytic inhibition might change local neuronal excitability or the synaptic activity of NTS neurons, as observed in this study.

The data of this study provides a better understanding of the complex interaction between putative astrocytes and neurons, via ATP release, at the NTS level, and contribute to explain, at least in part, the neurotransmission/neuromodulation mechanisms involving l-glutamate and ATP in the NTS neurons involved with the chemoreflex pathways (Braga et al. 2007; Accorsi-Mendonça et al. 2009). In addition to shedding lights on the role of astrocytes as an active player in the synaptic transmission at the NTS level, these findings may also have several important physiological and pathophysiological implications for a better understanding of the neuronal plasticity that may occur in acute and chronic hypoxic conditions.
